# Exploration of the ubiquitination-related molecular classification and signature to predict the survival and immune microenvironment in colon cancer

**DOI:** 10.3389/fgene.2024.1292249

**Published:** 2024-08-29

**Authors:** Ji-Zhong Xu, Tian-Qi Wan, Jin-Song Su, Jun-Min Song

**Affiliations:** Department of Colorectal Surgery, the First Affiliated Hospital of Zhengzhou University, Zhengzhou, China

**Keywords:** ubiquitination, molecular classification, prognosis, immune microenvironment, colon cancer, cell proliferation

## Abstract

**Background:**

Ubiquitination, a major post-translational modification, significantly impacts tumorigenesis, progression, and prognosis. This study aims to classify colon cancer at the molecular level and create a reliable signature using ubiquitination-related genes (URGs) to assess the immune microenvironment and prognosis.

**Methods:**

We employed non-negative matrix factorization to subtype colon cancer based on ubiquitination-related gene (URG) expression patterns. Quantitative scores for 28 immune cell infiltrates and the tumor microenvironment were computed using single-sample gene set enrichment analysis (ssGSEA) and the Estimate algorithm. Subtype feature genes were selected through Lasso logistic regression and SVM-RFE algorithm. The ubiquitination-related signature was constructed using univariate Cox, Lasso, and stepwise regression methods to categorize patients into high and low-risk groups. Validation included log-rank tests, receiver operating characteristic (ROC) analysis, decision curve analysis (DCA), and external dataset validation. Immune therapy response was compared using Tumor Immune Dysfunction and Exclusion (TIDE), Immunophenoscore (IPS), and submap analyses. Clinical variables and risk scores were integrated into an enhanced nomogram. The early diagnostic value of four URGs was confirmed via quantitative real-time polymerase chain reaction (qRT-PCR) and immunohistochemistry. The cell proliferation was assessed through colony formation, EdU staining, and xenograft tumorigenesis assays.

**Results:**

Prognostic ubiquitination-related genes (URGs) stratified patients into subtypes, revealing differences in survival, immune cell infiltration, and pathological staging. A signature of 6 URGs (ARHGAP4, MID2, SIAH2, TRIM45, UBE2D2, WDR72) was identified from 57 subtype-related genes. The high-risk group exhibited characteristics indicative of enhanced epithelial-mesenchymal transition, immune escape, immunosuppressive myeloid-derived suppressor cells, regulatory T cell infiltration, and lower immunogenicity. In contrast, the low-risk group demonstrated the opposite trend but showed a better response to CTLA4 checkpoint inhibitors. The predictive performance of the nomogram significantly improved with the integration of risk score, stage, and age. ARHGAP4 and SIAH2 exhibit promising early diagnostic capabilities. Additionally, WDR72 knockdown significantly inhibited CRC cell proliferation both *in vitro* and *in vivo*.

**Conclusion:**

Our developed ubiquitination-related signature and genes serve as promising biomarkers for colon cancer prognosis, immune microenvironment, and diagnosis.

## Introduction

Colorectal cancer (CRC) is a prevalent malignant tumor globally and is responsible for significant cancer-related mortality (Siegel et al., 2023a). In particular, China leads globally in both new cases and deaths associated with colorectal cancer (CRC) (Association, 2023). According to the 2020 Cancer Statistics Report of China, CRC ranked second in incidence and fifth in mortality among all malignancies, with 555,000 new cases and 286,000 deaths reported. Between 2000 and 2016, statistics show an average annual increase in CRC incidence of 2.4% among males and 1.2% among females in China (Yang et al., 2023). With the implementation of standardized diagnostic and treatment methods such as risk assessment combined with a two-step screening strategy based on colonoscopy and non-invasive examinations, the overall CRC mortality rate in China has decreased from 12.00 per 100,000 person-years in 1974 to 7.95 per 100,000 person-years in 2020, showing a slight downward trend (Xu et al., 2023). Despite the progress made in diagnosis and treatment, the prognosis of CRC remains unfavorable, with an approximate 5-year survival rate of 65% (Siegel et al., 2023b). The pathogenesis of CRC is characterized by intricate interactions between genetic and environmental factors, resulting in the aberration of diverse cellular mechanisms, including protein post-translational modifications (PTMs). One such PTM that has garnered significant attention in recent years is ubiquitination.

Ubiquitination is a highly conserved process that involves the conjugation of a small protein called ubiquitin to a target protein through covalent bonding. This modification is essential for the regulation of protein turnover, localization, and activity. The malfunction of the ubiquitin system has been associated with various diseases, such as cancer (Senft et al., 2018). In CRC, aberrant ubiquitination has been demonstrated to participate in tumor initiation, progression, and metastasis (Zhu et al., 2022). Recent studies have highlighted the potential of URGs as prognostic markers and therapeutic targets in CRC. One example is TRIM25, an E3 ubiquitin ligase that has been demonstrated to enhance the proliferation, invasion and oxaliplatin resistance of CRC cells (Zhou et al., 2021), while the deubiquitinase USP7 has been implicated in CRC epithelial mesenchymal transition (Basu et al., 2023). These findings indicate that targeting the ubiquitin system may be a promising treatment strategy for CRC. Ubiquitination has also been associated with the modulation of immune response in addition to its involvement in tumorigenesis (Liu et al., 2021). The development and progression of cancer are greatly influenced by the immune system, with the infiltration of immune cells into tumors playing a crucial role in determining patient prognosis (Lei et al., 2020). The presence of tumor-associated macrophages (Wang et al., 2021) and regulatory T cells (Aristin et al., 2022), for example, has been shown to correlate with poor prognosis in CRC. Therefore, comprehending the correlation between ubiquitination and the immune microenvironment could provide insights into the development of effective immunotherapies.

The applications based on genomic data has emerged as a promising approach to improve the clinical management of CRC. For example, a study identified a set of cell cycle-related periodic transcripts by analyzing high-resolution phase-lapsed cell-cycle-synchronized transcriptomes and successfully utilized them for predicting drug sensitivity in CRC (Li et al., 2022; Qiu et al., 2023). A 12-gene signature employed in the Oncotype DX Colon Cancer Assay anticipates the probability of chemotherapy advantages and the possibility of recurrence in patients with stage II colon cancer (Venook et al., 2013). Similarly, the ColoPrint ^®^ assay uses an 18-gene signature to forecast the outcome in patients with stage II and III colorectal cancer (Salazar et al., 2011). These multi-gene assays have been demonstrated to offer information regarding survival prognosis beyond that provided by traditional clinicopathological factors, such as tumor stage and grade (Xue et al., 2024; Zhong et al., 2022). Despite these advances, significant challenges remain in the development and clinical application of multi-gene prediction models in CRC. These include the heterogeneity of tumor, the need for large-scale validation studies, and the cost and accessibility of genomic testing. Nonetheless, with the increasing availability of genomic data and advances in computational biology, the use of multi-gene prediction models is likely to become increasingly widespread in the clinical treatment of CRC (Ahluwalia et al., 2021).

The current research explored the molecular classification and prognostic value of URGs in CRC, as well as their relationship with the immune microenvironment (Zhao et al., 2022). Using genomic data from large-scale cohorts, we developed a novel multi-gene signature based on URGs to predict the prognosis and immune microenvironment of CRC patients. Moreover, experimental validation was performed on the key genes identified by bioinformatics analysis and further explored their value in early diagnosis of CRC. The results of our study shed light on the potential of ubiquitination-related genes as prognostic markers and therapeutic targets in CRC, as well as the use of multi-gene prediction models in the clinical application.

## Materials and methods

### Data source and preprocessing

The study employed primary colorectal cancer patient samples from two cohorts. Gene expression and clinical data for the TCGA-COAD cohort (n = 424) were obtained from UCSC Xena website (https://xenabrowser.net/datapages/). For the TCGA-COAD dataset, the initially provided data in Fragments Per Kilobase Million (FPKM) underwent transformation to Transcripts Per Million (TPM). Another cohort GSE39582 (n = 573) was retrieved from the Gene Expression Omnibus (https://ncbi.nlm.nih.gov/geo/query/acc.cgi?acc=GSE39582) and the expression data was normalized using the limma R package. All data of the two cohorts underwent log2(x + 1) transformation, and potential batch effects were mitigated using the Combat algorithm within the SVA R package. For data consistency, samples with a survival duration of less than 30 days were excluded, and those exceeding 3,650 days were uniformly designated as 3,650 days, classifying them as alive for survival status assessment.

### Extraction of ubiquitination-related genes (URGs)

The URGs included in this study were retrieved from version 2.0 of the Integrated Annotations for Ubiquitin and Ubiquitin-like Conjugation Database (iUUCD) (http://iuucd.biocuckoo.org/) (Zhou et al., 2018). The database provides a comprehensive summary of data on ubiquitin-activating enzymes, ubiquitin-conjugating enzymes, ubiquitin-protein ligases, deubiquitinating enzymes, ubiquitin-binding domain-containing proteins, and ubiquitin-like domains. The database provides a total of 1360 URGs, while this study included 1299 URGs present in the TCGA-COAD and GSE39582 datasets (Supplementary Material S1).

### Molecular subtyping analysis in the TCGA-COAD cohort

Gene expression data matrix of the 1299 URGs were extracted from the TCGA-COAD cohort. Firstly, we conducted univariate Cox regression analysis to identify variables with *P*-value < 0.1, and then non-negative matrix factorization was performed with the NMF R package (Devarajan, 2008). The best subtyping was determined by the rank value corresponding to the maximum change in cophenetic value with K variation. The specific NMF parameters were set as rank = 2:6, method = “brunet”, nrun = 50, seed = 123,456. Then, the survival R package was used to perform analysis of OS and PFS for different subtypes. Then we applied Estimate algorithm to score the immune microenvironment and stromal microenvironment, and the GSVA R package’s ssgsea method was applied to quantify the infiltration degree of 28 immune cells according to the previous study (Charoentong et al., 2017). All scores were normalized to a 0–1 numerical distribution using the x = (x-min(x))/(max(x)-min(x)) function, and differences were analyzed using the Wilcoxon test.

### Identification of feature genes using machine learning

We employed machine learning techniques, specifically Lasso logistic regression and SVM-RFE, to select feature genes associated with the ubiquitination subtype. For Lasso logistic regression, we used the glmnet R package with 10-fold cross-validation to screen the feature genes corresponding to lambda.min. SVM-RFE, on the other hand, involved 5-fold cross-validation using the e1071 R package to perform feature variable selection and estimate generalization error with different feature combinations. The feature genes were selected based on the intersection genes obtained from these two methods, which were determined by selecting the minimum error.

### The establishment of the URGs-based prognostic model

TCGA-COAD was used as the training dataset while GSE39582 was employed as the validation dataset to construct a ubiquitination-related prognostic model. The initial variables are selected using the survival R package to screen for univariate Cox *P* values less than 0.01. The Lasso regression was employed to choose the variables at the time of lambda.min and the stepwise regression analysis was used to build a multi-gene Cox model which could calculate the patient’s risk score. The TCGA-COAD cohort was stratified into high-risk and low-risk groups based on the median risk score, and survival differences were analyzed using the Kaplan-Meier log-rank test, and the timeROC R package was used to draw ROC curves and evaluate model accuracy according to the AUC value. Clinical variables (age, gender, stage) are then incorporated and we conducted a multivariable Cox regression analysis to evaluate the risk score as an independent prognostic factor. The risk score and clinical features are integrated using the rms R package to plot the predictive survival line graph for 1, 3, and 5 years. The calibrate function is used to create a calibration curve. Finally, the decision curve analysis of multi-features in 5-year survival prediction is calculated with the ggDCA R package to compare the AUC values of the integrated line graph, risk score, and other clinical variables, and to calculate the net benefits ratio (Vickers and Elkin, 2006).

### Immune microenvironment and immunotherapy analysis

The mutation data in MAF format output by VarScan2 method in TCGA-LUAD project was downloaded from TCGA official website, and the maftools R package was employed to compute Tumor Mutation Burden (TMB). The Tumor Immune Dysfunction and Exclusion (TIDE) algorithm (http://tide.dfci.harvard.edu/) was utilized to predict immune checkpoint inhibitor responsiveness and calculate TIDE score. In addition, a submap algorithm from the GenePattern platform (https://cloud.genepattern.org/gp) was used to predict subtypes of immune therapy responsiveness (Ay et al., 2011). The Cancer Immunome Atlas (TCIA) database (https://tcia.at/) was employed to compute the Immunophenoscore (IPS). To identify enriched immune gene sets, Gene Set Enrichment Analysis (GSEA) was applied to 28 immune-related gene sets. The correlation between risk score and immune infiltration score was assessed using Pearson correlation analysis, and differences in immune cell infiltration between the high and low risk groups were compared using the Wilcoxon rank sum test.

### qRT-PCR

Forty-one pairs of cancer and paracancer pairs were extracted from the TCGA-COAD cohort for paired Wilcoxon differential test, and FDR less than 0.05 was considered as a potential diagnostic gene. Then, a total of thirty primary colon cancer tumors and paired paracancer samples were collected from untreated Stage IIA or Stage IIB colon cancer patients in our hospital. To verify the mRNA expression of genes incorporated in the model, we conducted qRT-PCR using the PrimeScript RT Reagent Kit and SYBR Premix Ex Taq, following the manufacturer’s instructions. The primer sequences were provided in Table 1. The CT values were acquired and analyzed using the 2^−ΔΔCt^ method.

**TABLE 1:** Primer sequences.

Symbol	Forward primer (5′-3′)	Reverse primer (5′-3′)
ARHGAP4	CTG​CGC​TTT​GAC​TAC​CAC​C	CAG​TGT​CGC​CTT​CAG​AGT​CT
SIAH2	CGC​CAG​AAG​TTG​AGC​TGC​T	TGG​TGG​CAT​ACT​TAC​AGG​GAA
TRIM45	AAC​TCA​GGC​AAG​ACT​CAC​TGC	CCC​TCG​GAT​GTC​CAC​TAC​TG
WDR72	GTG​GAG​AAG​GCT​ACA​CTT​CCT	CAA​TGC​ACA​TGC​AGT​TGA​TCC
GAPDH	CAG​CCT​CAA​GAT​CAT​CAG​CA	TGT​GGT​CAT​GAG​TCC​TTC​CA

### Immunohistochemistry

The tissue samples were subjected to deparaffinization and rehydration, and then underwent antigen retrieval via a 10 mM Na-Citrate buffer. To inhibit endogenous peroxidase, 0.3% H_2_O_2_ was applied for 15 min. After blocking with 10% goat serum for 30 min, primary antibodies (Table 2) were used for staining. Images were captured at ×100 and ×400 magnifications.

**TABLE 2:** Primary antibodies used in this study.

Symbol	Dilution	Catalog	Company
ARHGAP4	1:200	HPA001012	Sigma-Aldrich
SIAH2	1:100	PA5-105291	Invitrogen
TRIM45	1:150	MA5-26228	Invitrogen
WDR72	1:200	PA5-63780	Invitrogen
KI67	1:150	ab15580	Abcam
BCL2	1:1,000	ab182858	Abcam
GAPDH	1:500	ab8245	Abcam

### Cell culture and transfection

NCI-H716 were purchased from ATCC. To construct WDR72 knockdown cell lines, two shRNA sequences were synthesized and shRNA oligos were cloned into the pLKO.1 vector. The target sequences of WDR72 were: shRNA1 CAG​TTG​CTT​ACG​AAA​TGG​TAA, shRNA2 GCA​TCA​TTC​ATG​GAT​AGC​AAA.

### Clone formation assay

Cells were seeded in 6-well culture plates at a density of 5 × 10^2^ cells per well. The cells were incubated at 37°C with 5% CO2, and the medium was refreshed every 3–4 days. After approximately 2 weeks, the formed cell colonies were counted and analyzed.

### Western blot analysis

The total protein was extracted and electrophoresed on sodium dodecyl sulfate-polyacrylamide gels. Then, the gels were transferred to PVDF membranes. After blocking PVDF membranes with protein in 5% skim milk for 60 min, primary antibodies were applied to them at 4°C for 8 h. The chemiluminescent detection of protein blots was performed after adding the horseradish peroxidase (HRP)-conjugated secondary antibody.

### EdU assay

Cells were seeded in 96-well plates at approximately 1,000 cells per well. A fluorescence-based cell proliferation kit (BeyoClick™ EdU-488, China) was used according to the manufacturer’s instructions.

### Xenograft tumor mouse models

The CRC cells NCI-H716 were prepared by trypsinization and adjusted to a density of 1 × 10^7^ cells/mL. Four-week-old BALB/c nude mice received a subcutaneous injection of 100 μL containing 1 × 10^6^ cells in the hind leg. Tumor size was measured every 3 days until the average size reached 50–100 mm³.

## Results

Prognostic ubiquitination-related genes (URGs) can divide patients with colon cancer into two molecular subtypes.

**FIGURE 1:**
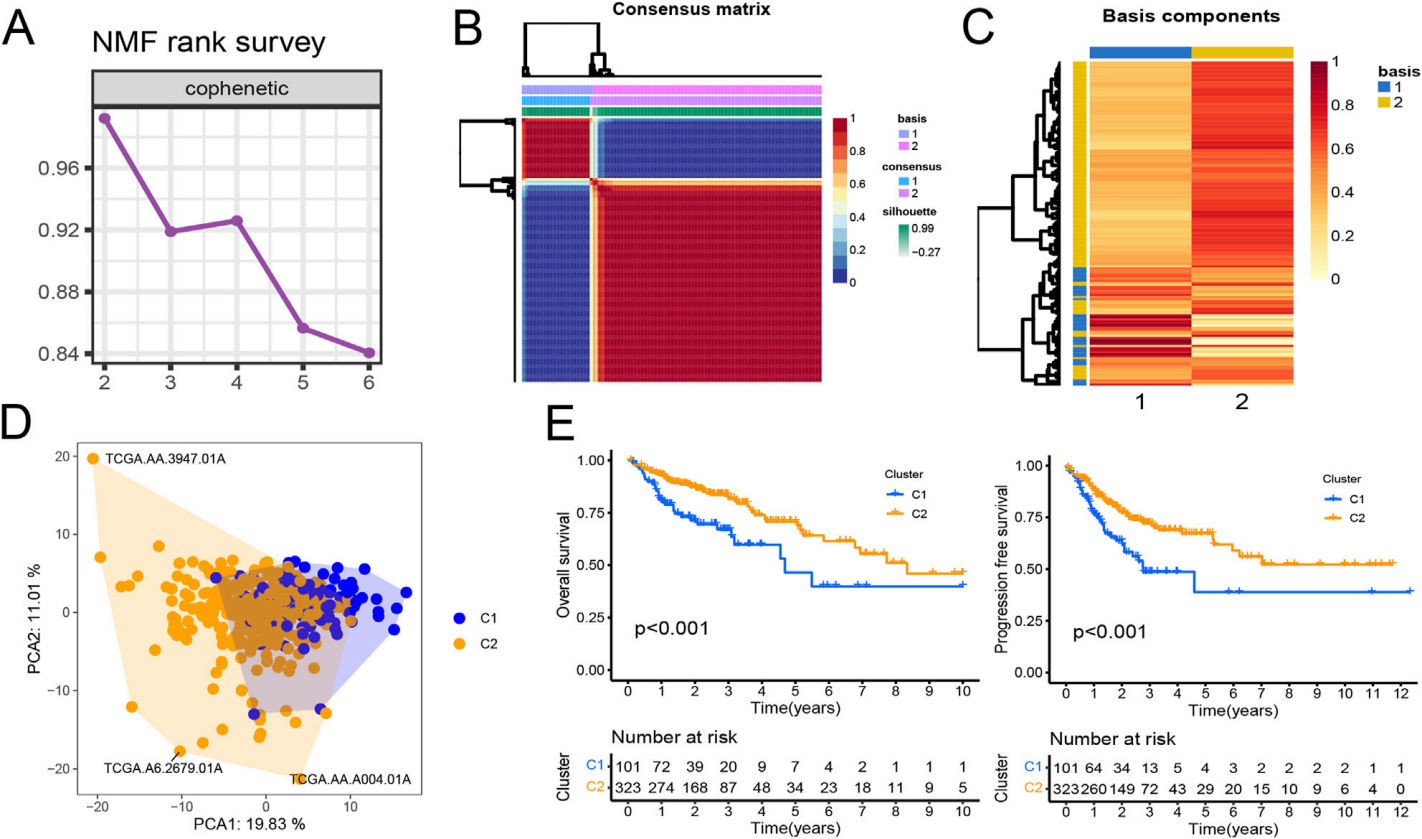
Prognostic ubiquitination-related genes (URGs) can divide colorectal cancer patients into two subtypes. **(A)** The trend of cophenetic value changes as K increases from 2 to 6 by NMF analysis. **(B)** Consensus matrix when k = 2. **(C)** The basis components heatmap when k = 2. **(D)** Principal component analysis of C1 and C2 clusters. **(E)** Survival (OS and PFS) analysis of C1 and C2 clusters in the TCGA-COAD cohort. Log-rank test.

**FIGURE 2:**
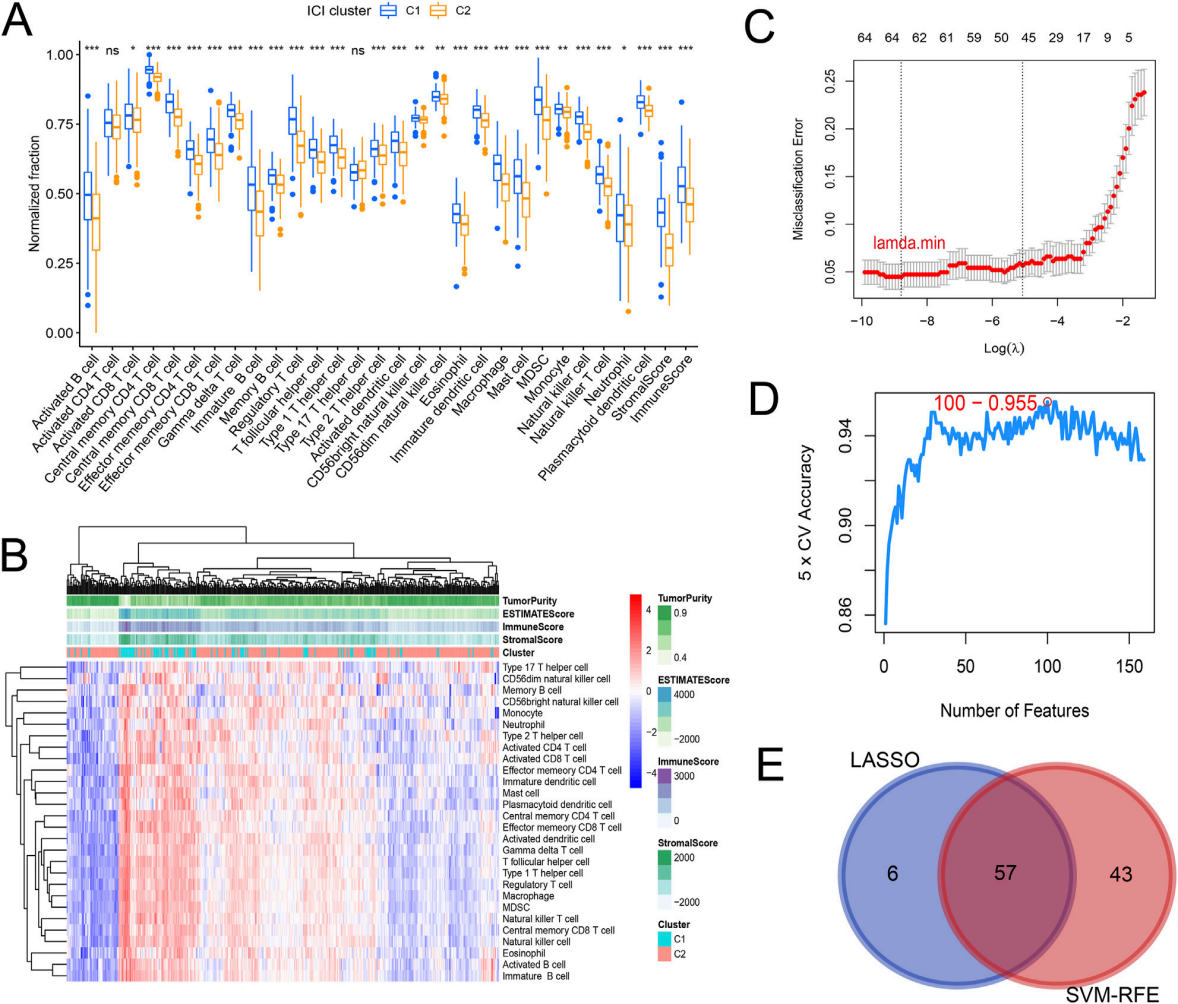
Differences in immune cell infiltration and screening of subtype-related characteristic genes. **(A)** Boxplot showing differences in immune cell infiltration and tumor microenvironment. **(B)** Heatmap showing the differences in immune cell infiltration and tumor microenvironment. It can be observed that C1 subtype is generally immune activated. **(C)** Lasso logistic regression screening 63 specific genes corresponding to the minimum lambda value through 10-fold cross-validation. **(D)** 100 genes with minimum error were selected using SVM-RFE and 5-fold cross-validation. **(E)** Venn diagram determined 57 intersected genes.

Gene expression data matrix of the 1299 URGs were extracted from the TCGA-COAD cohort. Firstly, we conducted univariate Cox regression analysis to identify candidate variables. A total of 159 univariate Cox proportional hazards regression models were screened based on a *P*-value of less than 0.1. Then, non-negative matrix factorization (NMF) analysis was performed based on the gene expression data matrix of the identified survival-related 159 URGs with parameters “rank = 2:6, method = “brunet”, nrun = 50, seed = 123,456”(Devarajan, 2008). The optimal number of clusters was determined by identifying the values of k where the magnitude of the cophenetic correlation coefficient started to decline (Figure 1A) (Brunet et al., 2004). As a result, two subtypes (C1 and C2) were identified. When the rank was set to 2, the boundary of the consensus matrix heat map was clearly defined, indicating a stable and robust clustering of samples (Figure 1B). The Basismap function was used to draw the basismatrix, and the gene clustering on each basis could be visualized (Figure 1C). Unlike Principal Component Analysis (PCA), which is a linear dimensionality reduction method, NMF preserves local features of each factor with equal weights after matrix decomposition, making it robust to noise and outliers compared to methods relying on the covariance matrix. Therefore, the effect of PCA method processing the same data matrix of the survival-related 159 URGs in distinguishing the two subtypes is not very significant (Figure 1D). The Kaplan-Meier curve showed that the C1 subtype was significantly (*P* < 0.001) worse than the C2 subtype in both Overall Survival (OS) and Progression-Free-Survival (PFS) (Figure 1E). Subsequently, based on the gene expression matrix of the TCGA-COAD cohort, we quantified the ImmuneScore and StromalScore for each sample using the Estimate algorithm (Yoshihara et al., 2013). In order to assess the differences in various types of immune cells, we quantified the immune cell infiltrations of the 28 immune cell types based on the gene signatures trained from a previous study ( Supplementary Material S2) (Charoentong et al., 2017) with the ssGSEA method from the GSVA R package (Hänzelmann et al., 2013). The estimate results demonstrated that the StromalScore and ImmuneScore of the C1 subtype were significantly higher. Among all 28 types of immune cells, we found that infiltration levels of as many as 26 types were significantly elevated in the C1 subtype (Figures 2A, B), suggesting that survival-related URGs was believed to have a significant impact on the immune microenvironment of CRC. Chi-square statistical analysis of clinical variables was performed for both subtypes using the chisq.test function. The average age of the C1 subtype was significantly lower than that of the C2 subtype, and a higher proportion of patients with stage III and IV, T3-4 tumors, and lymph node metastasis was found in the C1 subtype. No statistical differences were found in gender or tumor metastasis (Table 3).

**TABLE 3:** The clinical characteristics between the C1 and C2 subtypes.

Variables	Group	Total	Cluster1	Cluster2	*P*-value
Age	≤65	180 (42.45%)	53 (52.48%)	127 (39.32%)	0.0265
	>65	244 (57.55%)	48 (47.52%)	196 (60.68%)	
Gender	Female	195 (45.99%)	50 (49.5%)	145 (44.89%)	0.4854
	Male	229 (54.01%)	51 (50.5%)	178 (55.11%)	
Stage	Stage I	72 (16.98%)	10 (9.9%)	62 (19.2%)	0.0014
	Stage II	160 (37.74%)	29 (28.71%)	131 (40.56%)	
	Stage III	121 (28.54%)	41 (40.59%)	80 (24.77%)	
	Stage IV	60 (14.15%)	19 (18.81%)	41 (12.69%)	
	unknow	11 (2.59%)	2 (1.98%)	9 (2.79%)	
pathologic_T	T1	10 (2.36%)	1 (0.99%)	9 (2.79%)	0.0023
	T2	75 (17.69%)	8 (7.92%)	67 (20.74%)	
	T3	289 (68.16%)	72 (71.29%)	217 (67.18%)	
	T4	49 (11.56%)	19 (18.81%)	30 (9.29%)	
	unknow	1 (0.24%)	1 (0.99%)	0 (0%)	
pathologic_N	N0	248 (58.49%)	40 (39.6%)	208 (64.4%)	<0.0001
	N1	101 (23.82%)	32 (31.68%)	69 (21.36%)	
	N2	75 (17.69%)	29 (28.71%)	46 (14.24%)	
pathologic_M	M0	314 (74.06%)	69 (68.32%)	245 (75.85%)	0.1455
	M1	60 (14.15%)	19 (18.81%)	41 (12.69%)	
	unknow	50 (11.79%)	13 (12.87%)	37 (11.46%)	

### Identification of feature genes associated with ubiquitination subtype using machine learning

To determine characteristic genes associated with tumor typing, we employed a joint screening approach using Lasso logistic regression and SVM-RFE based on the gene expression matrix of the identified 159 prognostic URGs in TCGA-COAD cohort and the subtype information of each sample. We inputted the expression profiles of 159 prognosis-related URGs from the TCGA-COAD cohort and subtype classification information of samples. Using the glmnet R package for lasso logistic regression through 10-fold cross-validation, we identified 63 URGs that are more important for classification when the misclassification error is minimized (Figure 2C). SVM-RFE method was used to select 100 genes with the least error using the e1071 R package and 5-fold cross-validation (Figure 2D). The final set of genes was obtained by intersecting the results from the two methods, resulting in 57 genes (Figure 2E).

### The establishment of the URGs-based prognostic model

**FIGURE 3:**
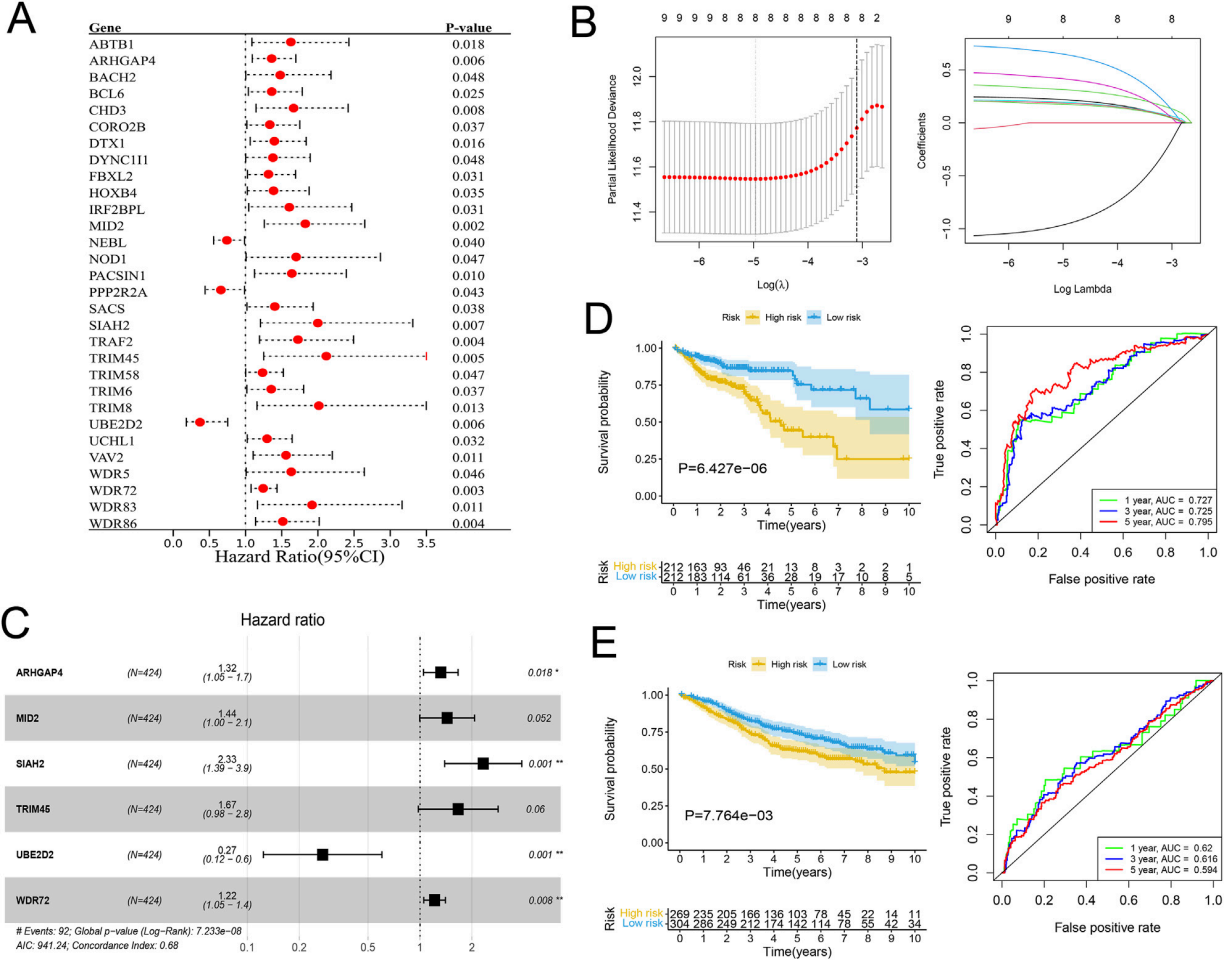
Establishment of the URGs-based prognostic model. **(A)** Forest plot of the Hazard ratios of the prognostic URGs through univariate Cox regression analysis. **(B)** Lasso regression analysis. Left: The curve of partial-likelihood deviance changing with Log(λ). A smaller value indicates a better model fit. Right: The curve of regression coefficients of each variable with the change of Log(λ). **(C)** Forest plot of the URGs-based prognostic model through stepwise regression analysis. **(D, E)** The KM survival curves and ROC curves for the training and validation cohorts separately.

**FIGURE 4:**
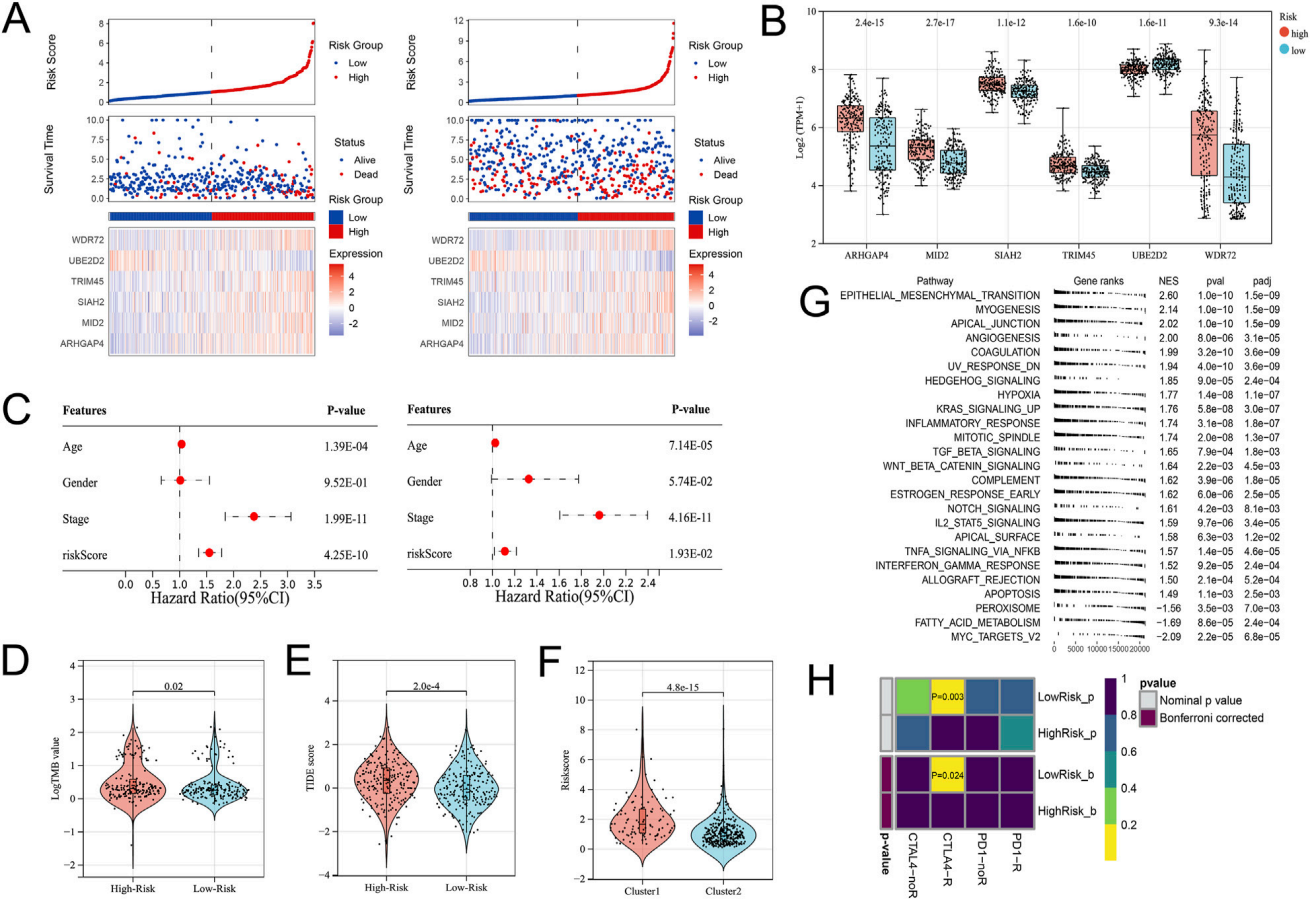
Differential gene expression patterns and immune therapy response in high- and low-risk groups. **(A)** The variation trend of survival time, survival state and gene expression with the risk score. **(B)** Boxplot showing the expression differences of the 6 URGs in the high- and low-risk groups. **(C)** Multivariate Cox analysis results of the training (Left) and validation (Right) cohorts indicated the risk score was an independent prognostic factor. **(D)** The TMB value of the High-risk group was higher than that of the Low-risk group. **(E)** The TIDE score significantly increased in the High-risk group. **(F)** The High-risk group’s risk score was significantly higher in the C1 subtype. **(G)** GSEA analysis. **(H)** Submap analysis showed that the Low-risk group might be more sensitive to the CTLA4 inhibitor (Bonferroni-corrected *P* = 0.024).

In view of the significant difference in survival prognosis between the two subtypes, we continued to reduce the dimension of 57 phenotypic genes to construct a polygenic cox prognostic model. Firstly, the URGs were identified by the univariate cox analysis. In order to simplify the model, 9 genes with *P* < 0.01 were included as initial variables (Figure 3A). Then, the 8 variables corresponding to lambda were further screened by lasso regression (Figure 3B). Finally, a 6-gene cox model was constructed by stepwise regression to calculate patients’ risk scores, named URGS in this study. Risk score = 0.278*ARHGAP4 + 0.363*MID2 + 0.845*SIAH2 + 0.511*TRIM45 - 1.300*UBE2D2 + 0.196*WDR72. Hazard ratio values of genes in the model and *P* values of multivariate Cox analysis are shown in the forest map (Figure 3C). Using the median risk score of 1.027 as the threshold, patients were stratified into high and low-risk groups in the training set. The survival analysis using Kaplan-Meier curve and Receiver Operating Characteristic (ROC) curve demonstrated significant differences in overall survival (OS) between high and low-risk groups with a log-rank test *P*-value of 6.427e-06 in the training set. Moreover, the predicted OS at 1, 3, and 5 years yielded AUC values of 0.727, 0.725, and 0.795, respectively (Figure 3D). In the external verification set, log-rank test *p* = 7.764e-03, and AUC values were 0.620, 0.616 and 0.594, respectively (Figure 3E). The variation trend of survival time, survival state and gene expression with risk score was shown (Figure 4A). UBE2D2 expression showed a gradual decrease trend with the increase of risk value, while the other 5 genes showed the opposite trend, which was also confirmed by Wilcoxon differential analysis results (Figure 4B). A multivariate Cox regression analysis was performed on age, gender, stage, and the risk score. The results showed that the risk score was an independent prognostic factor for OS in both cohorts, as the *P* values were less than 0.05 (Figure 4C).

### URGS is significantly associated with immune microenvironment and immunotherapy

**FIGURE 5:**
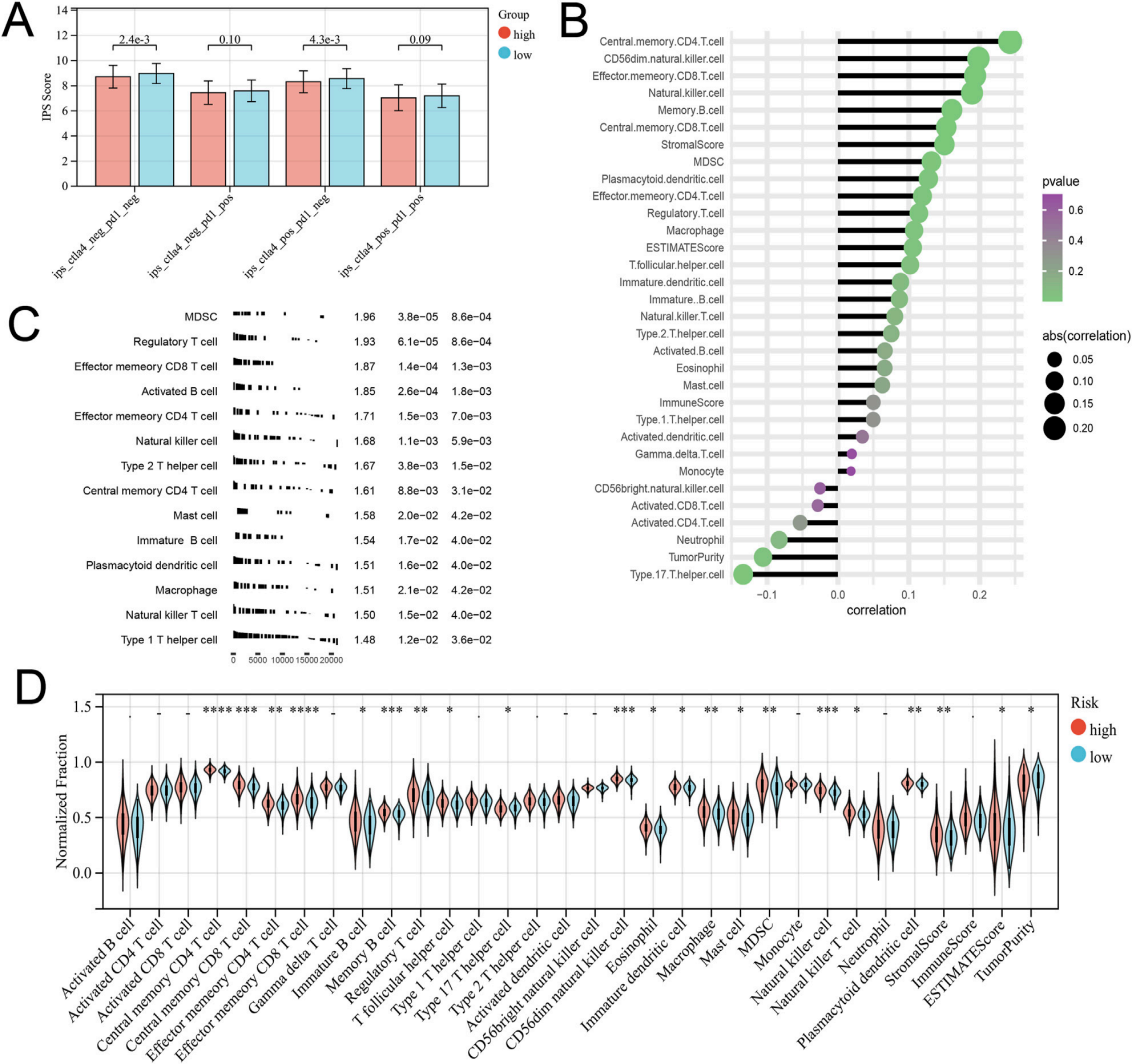
Different immune infiltration patterns between high and low-risk groups. **(A)** The Differential analysis of the IPS score representing immunogenicity among the four subtypes. **(B)** Correlation between Risk Score and Immune Cell Infiltration. **(C)** GSEA analysis with the background of immune cell infiltration gene set. **(D)** Differences in immune cell infiltration and tumor microenvironment scores between high-risk and low-risk groups.

**FIGURE 6:**
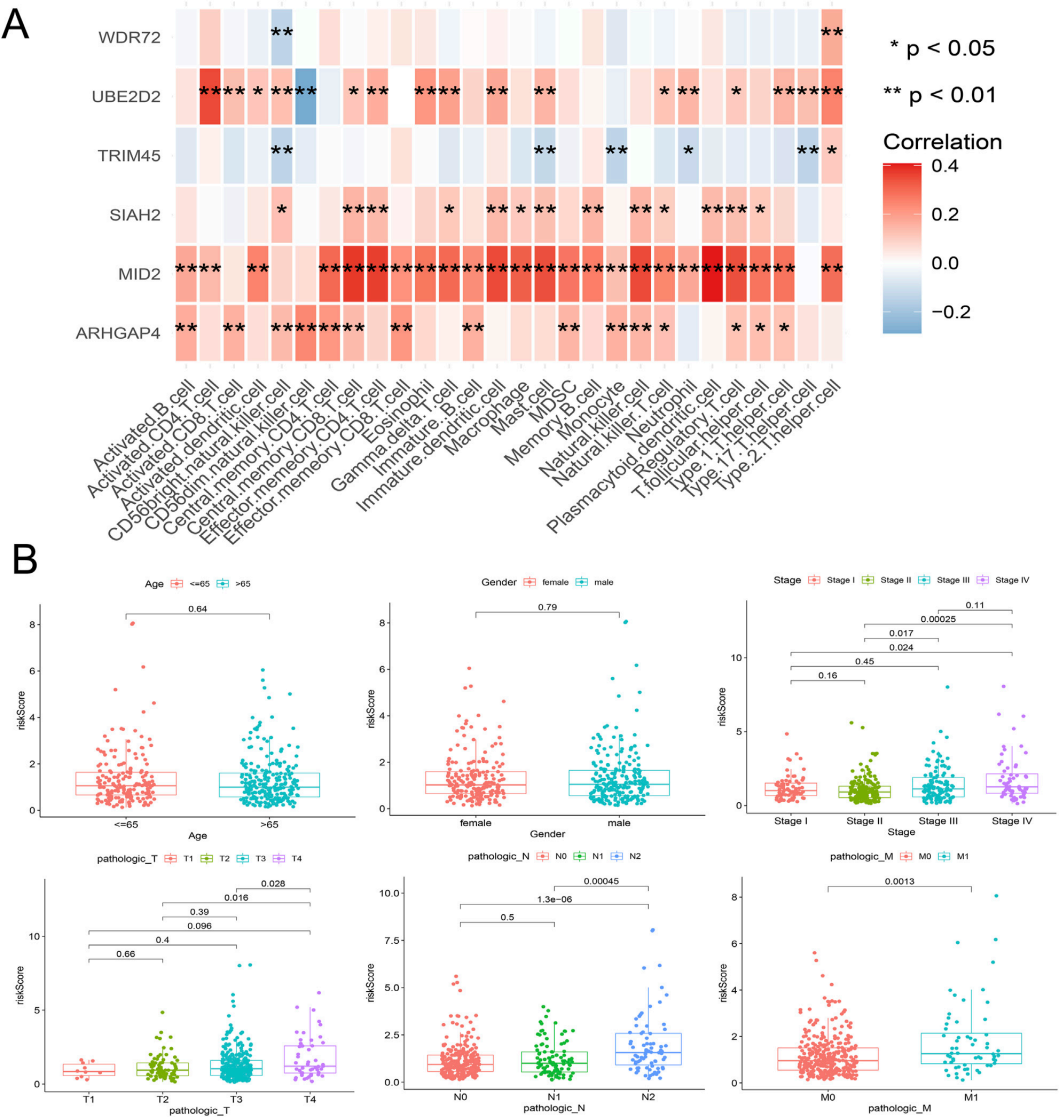
Correlation analysis between the model’s internal genes and immune cell infiltration **(A)**, as well as the correlation between the risk score and clinical characteristics **(B)**.

From the previous results, it is known that there are significant differences between the immune microenvironments of two subtypes. We studied the relationship between the risk score and TMB, TIDE, immune cell infiltration, and response to immunotherapy in the TCGA-COAD cohort. The findings indicated that the High-risk group possessed a TMB value that slightly exceeded that of the Low-risk group (Figure 4D). However, there was a notable rise in the TIDE score within the High-risk group, indicating a more severe degree of immune escape (Figure 4E). The TIDE algorithm indicated that the Low-risk group potentially holds greater potential for immunotherapy responsiveness in contrast to the High-risk group. Furthermore, the High-risk group demonstrated a considerably elevated risk score within the C1 subtype as compared to the C2 subtype (Figure 4F). GSEA analysis of Hallmark gene sets showed that the high-risk group exhibited a substantial enrichment of Inflammatory response and other pathways such as EMT, Myogenesis, and Apical junction. The Low-risk group showed significant enrichment of MYC targets, Fatty acid metabolism, and Peroxisome (Figure 4G). According to the Submap analysis, the Low-risk group demonstrated a potentially greater susceptibility towards the CTLA4 inhibitor (*P* = 0.024, Bonferroni-corrected) (Figure 4H). All four subtypes demonstrated considerably heightened immunogenicity in the Low-risk group as the IPS score notably exceeded (Figure 5A). The Pearson correlation analysis did not reveal a strong correlation between the risk score and immune cell infiltration, due to the relatively small correlation coefficients (Figure 5B). The GSEA findings demonstrated that fourteen out of twenty-eight immune cell gene sets exhibited significant enrichment in the High-risk group. Of these, the three most prominently enriched gene sets within this subgroup were those of myeloid-derived suppressor cells (MDSC), regulatory T cells, and effector memory CD8 T cells (Figure 5C). Moreover, the Wilcoxon test findings displayed a notable elevation in the levels of infiltration of these three immune cells, within the High-risk group in comparison to the Low-risk group (Figure 5D). The roles of the first two immune cells in immune suppression and promoting tumor progression have been widely reported. Among the six genes in the model, MID2, ARHGAP4, SIAH2, and UBE2D2 showed a strong positive correlation with immune cell infiltration, while WDR72 and TRIM45 showed weak correlation with most immune cell infiltrates (Figure 6A).

### Clinical correlation analysis of URGS and construction of prognostic nomogram

**FIGURE 7:**
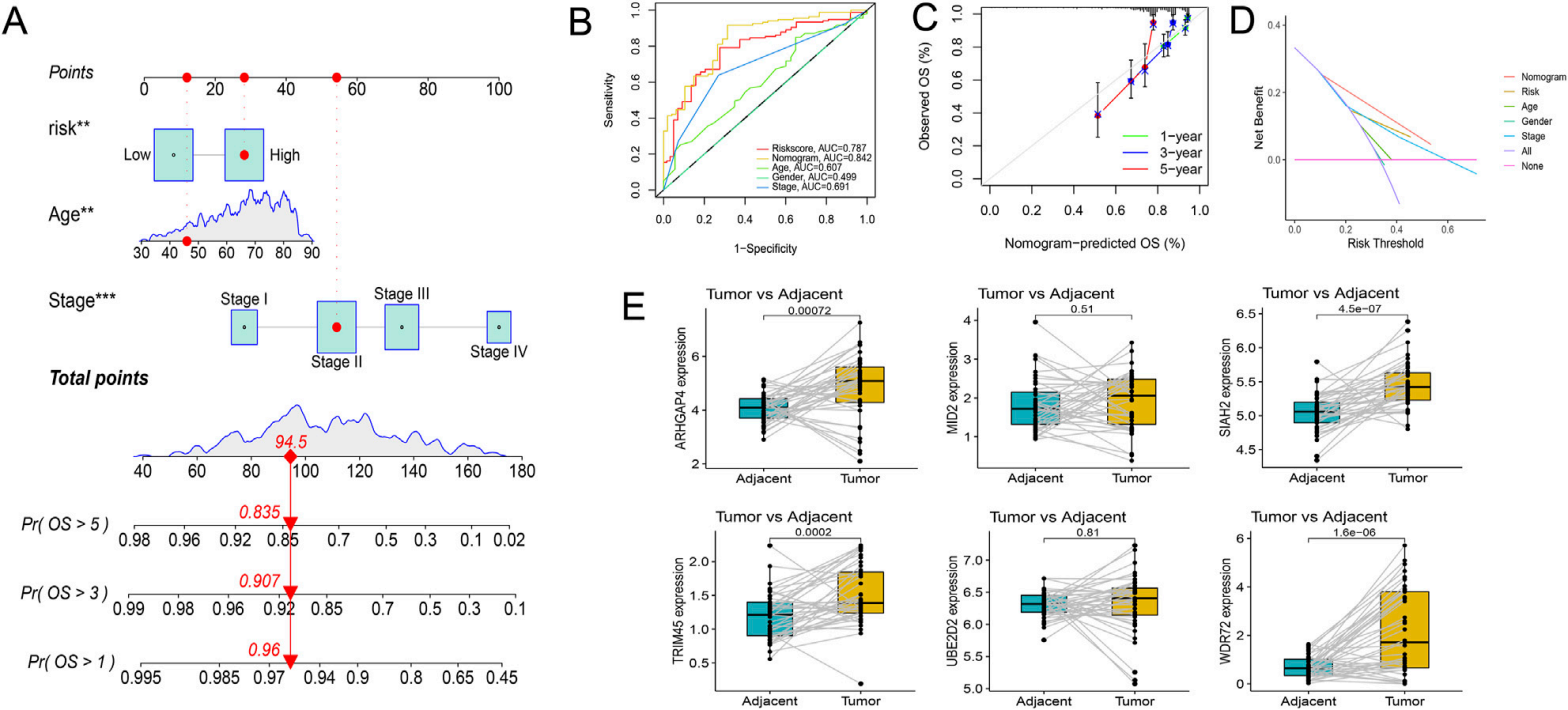
Exploration of the prognostic nomogram. **(A)** The nomogram including Age, Stage, and risk score. **(B)** Multi-indicator ROC curve indicates the nomogram’s prediction performance is much better than other clinical variables. **(C)** The calibration curves. **(D)** The DCA decision curves. **(E)** Differential gene expression analysis in paired samples from the TCGA-COAD cohort.

Risk scores were compared across all subgroups using a Wilcoxon test, which revealed no significant difference in risk score within the Age and Gender subgroups, but showed an increasing trend with the rise of Stage and T stage, particularly in Stage IV and T4 patients. In terms of tumor metastasis, stage N2 and stage N0-N1, and stage M1 showed significantly higher risk values than stage M0 (Figure 6B). This outcome corresponds with the findings of the GSEA enrichment analysis, which exhibited a significant enrichment of the EMT pathway within the high-risk group. Subsequently, based on independent prognostic analyses, Age, Stage, and the risk score were included in the TCGA-COAD cohort to construct a nomogram (Figure 7A). The multi-indicator ROC curves indicated that the Nomogram’s 5-year OS AUC prediction achieved 0.842, which is much higher than other clinical variables and significantly improved compared to the risk score’s AUC value of 0.787 (Figure 7B). Calibration curves also show the prediction efficiency of this nomogram is close to the ideal state (Figure 7C). The DCA decision curve further confirmed the integrated nomogram has the highest net benefit rate (Figure 7D). The difference analysis of 41 paired samples of TCGA-COAD showed that the mRNA levels of ARHGAP4, SIAH2, TRIM45, and WDR72 were significantly upregulated in the tumor group, while MID2 and UBE2D2 showed no statistical difference (Figure 7E).

### Experimental validation indicated ARHGAP4 and SIAH2 had high diagnostic values

**FIGURE 8:**
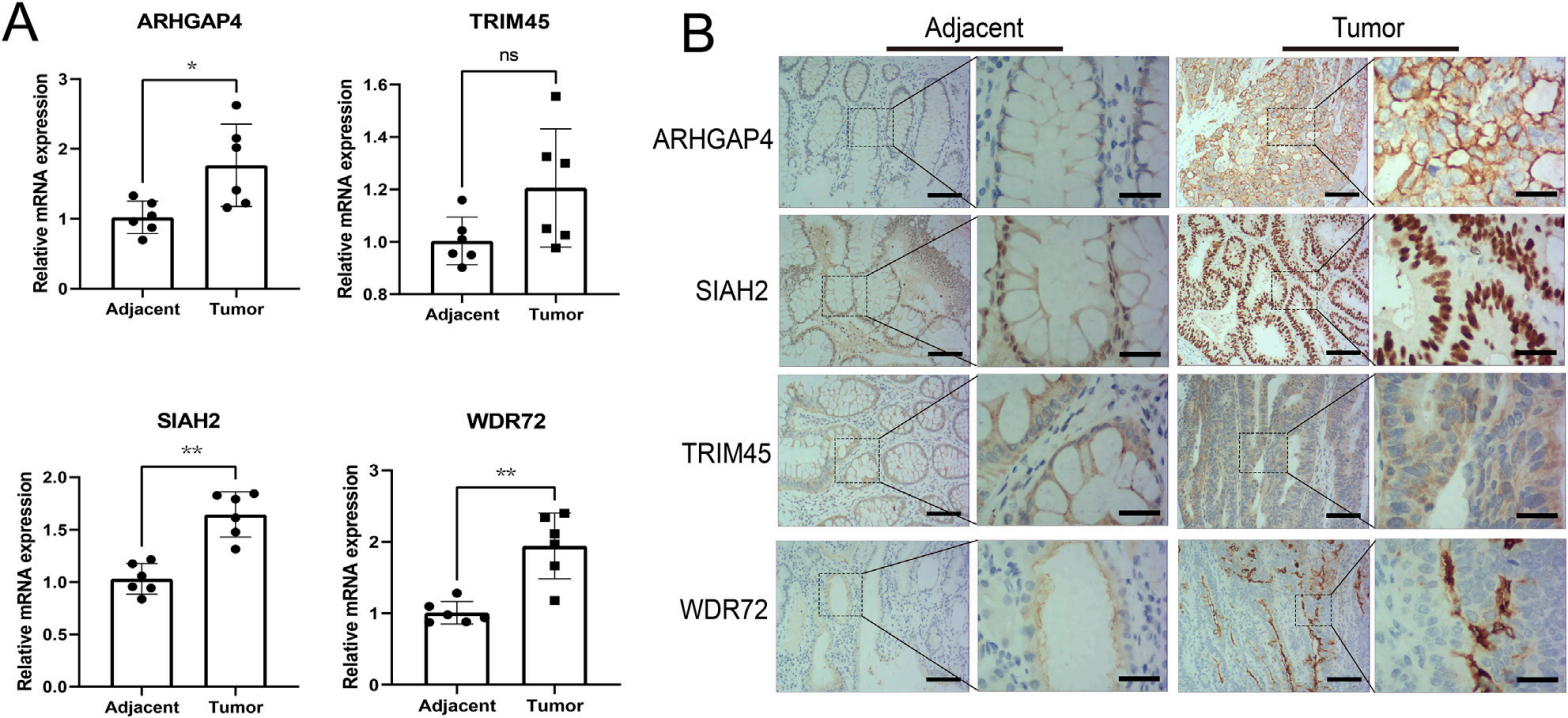
Experimental validation the diagnostic values of the identified URGs. **(A)** Differential analysis of mRNA expression of four genes in early-stage colon cancer patient samples by qRT-PCR. **(B)** Representative images from immunohistochemical assays at the protein level were captured at ×100 and ×400 magnifications. Scale bar: 50 μm and 15 μm separately. *P < 0.05, **P < 0.01, ns: no significance.

qRT-PCR was conducted to validate the mRNA expression of four differentially expressed genes in untreated Stage IIA colon cancer patients in our hospital. The results revealed significant upregulation of ARHGAP4, SIAH2, and WDR72 in the tumor group, while TRIM45 did not show a statistical difference (Figure 8A). Immunohistochemical assays confirmed these findings, with ARHGAP4 and SIAH2 showing optimal diagnostic potential at the protein level (Figure 8B).

### Knockdown of WDR72 inhibits the proliferation of CRC cells *in vivo* and *in vitro*

**FIGURE 9:**
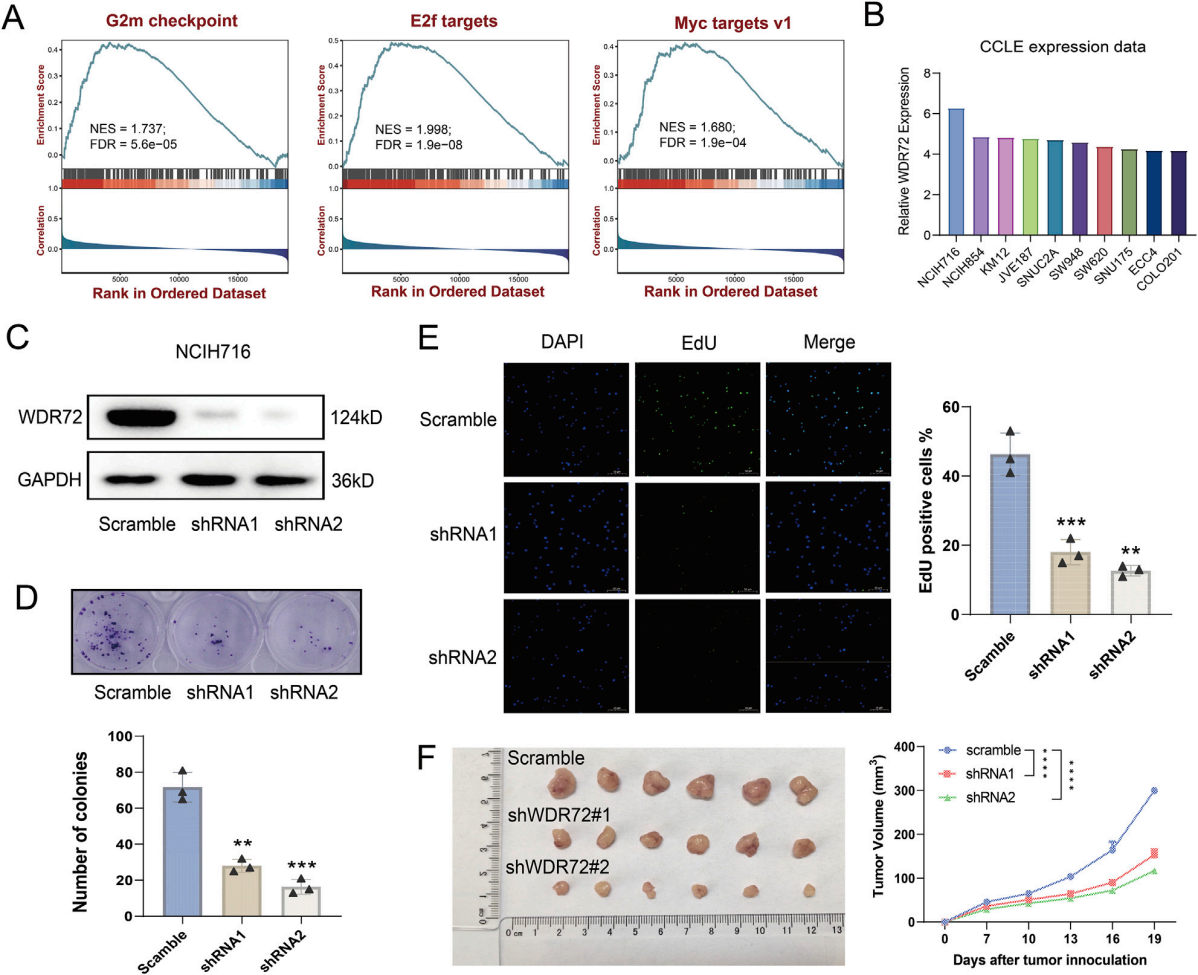
Knockdown of WDR72 inhibits the proliferation and promotes the apoptosis of CRC cells. **(A)** GSEA analysis indicates that WDR72 is positively correlated with E2F targets, G2M checkpoint, and Myc targets v1 pathways. NES: normalized enrichment score. FDR: false discovery rate. **(B)** The top 10 CRC cell lines with the highest relative mRNA expression levels of WDR72 according to the CCLE database. **(C)** Western blot analysis was used to detect the protein expression in WDR72 knockdown NCI-H716 cell lines. **(D)** Colony formation assays were conducted to assess the proliferation capacity of WDR72 knockdown cell lines, with each group including three replicates. **(E)** EdU staining assays were performed to detect the proportion of proliferative cells following WDR72 knockdown, with the percentage of EdU-positive cells representing the level of apoptosis. Each group included three replicates. Scale bar = 50 μm. **(F)** Tumor growth in nude mice was monitored after subcutaneous injection of WDR72 knockdown NCI-H716 cells, with tumor volumes measured every 3 days starting from day 7 (n = 6/group). Data are presented as mean ± SD. Statistical significance was determined using Student’s t-test or two-way ANOVA with Tukey’s multiple comparison test, **P < 0.01, ***P < 0.001, ****P < 0.0001.

Among the identified URGs, WDR72 is significantly overexpressed in CRC tissue samples, and its function has not yet been reported. As shown in Figure 6A, WDR72 is not correlated with the infiltration of most immune cells. Based on transcriptomic data from the TCGA-COAD cohort, we performed GSEA analysis on WDR72. The results indicated that WDR72 is positively correlated with E2F targets, G2M checkpoint, and Myc targets v1 pathways, suggesting that WDR72 may promote cell proliferation in CRC (Figure 9A). Subsequently, we selected the NCI-H716 cell line, which exhibits the highest expression of WDR72 among colorectal adenocarcinoma cell lines according to the CCLE database (Figure 9B), for WDR72 gene knockdown. The knockdown efficiency was verified by Western blot (Figure 9C). Colony formation assays and EdU immunofluorescence staining indicated WDR72 knockdown significantly inhibited the *in vitro* proliferation of NCI-H716 cells (Figures 9D, E). We then conducted a xenograft tumor experiment using the NCI-H716 cell line in nude mice, which demonstrated that WDR72 knockdown significantly suppressed the subcutaneous tumor formation ability of CRC cells (Figure 9F). These results suggest that WDR72 knockdown can inhibit CRC cell proliferation, indicating its potential as a therapeutic target. Further investigation into the molecular mechanisms and other functions of WDR72 will be the focus of future research.

## Discussion

Colorectal cancer (CRC) is a prevalent form of malignancy worldwide, with a significant impact on patient morbidity and mortality. The development and progression of CRC involve a complex interplay of genetic and epigenetic alterations, as well as alterations in various signaling pathways (Grady et al., 2021). Among these, ubiquitination has emerged as a crucial player in tumorigenesis, development, and prognosis (Chen et al., 2020). Also, CRC is a highly heterogeneous disease, and the distinctiveness originates not solely from genetic disparities or molecular heterogeneity but also by heterogeneity of the immune microenvironment (Zhang et al., 2020a). The immune microenvironment of CRC is composed of various immune cell types, such as T cells, B cells, dendritic cells (DCs), macrophages, and myeloid-derived suppressor cells (MDSCs), among others. Despite the presence of immune cells, the immune microenvironment of CRC is generally considered to be immunosuppressive, creating a barrier to effective anti-tumor immunity (Zhang et al., 2020b). This immunosuppression is largely attributed to the recruitment and activation of immunosuppressive cell populations such as MDSCs and regulatory T cells (Tregs). These cells create a pro-tumorigenic environment by suppressing effector T cell activity and promoting tumor growth and invasion (Ooki et al., 2021). In contrast, the presence of CD8^+^ T cells, NK cells, and mature DCs has been associated with a favorable prognosis in CRC (Jin et al., 2020). The heterogeneity of the immune microenvironment in CRC poses a great challenge. Different regions within a single tumor can exhibit distinct immune profiles, and the immune microenvironment can also vary between primary tumors and metastases (Liu Y. et al., 2022). Thus, a comprehensive understanding of the immune microenvironment in CRC is necessary to identify optimal therapeutic targets and improve treatment outcomes.

Ubiquitination pertains to a mechanism in which a substrate protein gets tagged with ubiquitin, a minute regulatory protein, leading to changes in the protein’s activity, stability, or localization (van Wijk et al., 2019). Aberrant ubiquitination has been implicated in various cancers leading to dysregulation of critical signaling pathways and alterations in the tumor microenvironment (Han et al., 2022). Specifically, ubiquitination can affect the tumor microenvironment, which in turn impacts the response to immunotherapy (Hu et al., 2021).

In this study, molecular subtyping based on ubiquitination-related genes (URGs) has emerged as a promising approach to predicting the prognosis and immune microenvironment of CRC. By using non-negative matrix factorization and other analytical techniques, we have identified URG-based molecular subtypes that correlate with survival prognosis, immune cell infiltration, and pathological staging. Furthermore, the construction of an effective ubiquitination-related signature based on six URGs (ARHGAP4, MID2, SIAH2, TRIM45, UBE2D2, WDR72) has allowed for the classification of patients into high and low-risk groups, with significant differences in immune cell infiltration, immunogenicity, and response to immunotherapy. Moreover, ARHGAP4, SIAH2, and WDR72 have demonstrated potential for early diagnosis of CRC.

ARHGAP4 belongs to the RhoGAP family of proteins and plays a crucial role in regulating Rho GTPase activity. Unfortunately, there have been few research advances on ARHGAP4 in the field of CRC and even in the entire field of tumor research. A bioinformatic study demonstrated upregulation of ARHGAP4 expression in CRC tissues and established a significant correlation between its overexpression and adverse prognosis (Fu et al., 2022). In another study, ARHGAP4 was identified as a potential oncogene in gastric cancer, and its high expression was significantly associated with poor prognosis (Sera et al., 2022). A fundamental study showed that ARHGAP4 is a novel regulatory factor of the HDAC2/β-catenin pathway, which inhibits β-catenin activation by interacting with and ubiquitinating HDAC2, and regulates cell invasion and migration of pancreatic cancer as well as the downstream effectors MMP2 and MMP9 expression *in vitro* (Shen et al., 2019). Therefore, ARHGAP4 has been proposed as a potential therapeutic target for CRC treatment.

MID2 is a constituent of the tripartite motif (TRIM) protein gene family. In breast cancer, it was observed that MID2 expression exhibited a notable upregulation and a positive correlation with stage, Ki67 protein expression, and poor prognosis, promoting cancer cell proliferation (Wang et al., 2016). Another study showed that MID2 mainly regulates cell division by ubiquitinating and degrading the microtubule-associated protein astrin, which may be an important pathway for promoting cancer cell proliferation (Gholkar et al., 2016). However, there have been no reports on MID2 in the field of CRC.

SIAH2 is an E3 ubiquitin ligase. One recent study has shown that the activation of the PI3K/AKT signaling pathway by SIAH2 can augment the proliferation and invasion of CRC cells (Hu et al., 2022). Additionally, SIAH2 has been proposed to be involved in the regulation of cancer stem cells and in orchestrating the response to hypoxia. It has been established that the Siah2-PHD3-HIF-1α axis serves to augment colon cell proliferation and the CSC population (Liu Q. et al., 2022).

TRIM45 is another URG identified in the signature developed by the study mentioned above. So far, there have been no reports in the field of CRC. Our findings concur with one bioinformatic study in hepatocellular carcinoma that has established a significant correlation between TRIM45 expression and adverse prognosis (Wu et al., 2022). However, TRIM45 has also been found to be a new tumor suppressor that enhances the stability and activation of p53 in glioma (Zhang et al., 2017). In our study, no significant variation was noted in the protein expression levels of TRIM45 in early-stage colon cancer tissues.

UBE2D2 is a member of the ubiquitin-conjugating enzyme (E2) family, which plays a protective role in our study. To date, there is no research on it in the field of oncology.

WDR72 displayed a considerable upregulation at the transcriptional and protein levels within the tumor tissues and showed a correlation with unfavorable prognosis in this study. However, there are few reports on WDR72 in tumors. It has been reported that the activation of the AKT/HIF-1α signaling pathway by WDR72 can lead to an increase in the stem-like properties of lung cancer stem cells (Ouyang et al., 2022). In our study, we found that knocking down WDR72 inhibited the proliferation of CRC cells and promoted their apoptosis through bioinformatics analysis and *in vitro* and *in vivo* gene function experiments.

Taken together, the investigation of the role of ubiquitination in CRC has highlighted the importance of post-translational modifications in cancer development and prognosis, as well as the critical role of the tumor microenvironment in cancer progression and therapy response. The identification of URG-based molecular subtypes and the construction of effective ubiquitination-related signatures have provided novel biomarkers for the prediction of prognosis, immune microenvironment, and response to immunotherapy in CRC.

## Data Availability

The datasets presented in this study can be found in online repositories. The names of the repository/repositories and accession number(s) can be found in the article/Supplementary Material.
